# Hybridization of graphene-gold plasmons for active control of mid-infrared radiation

**DOI:** 10.1038/s41598-024-57216-6

**Published:** 2024-03-20

**Authors:** Matthew D. Feinstein, Euclides Almeida

**Affiliations:** 1 Department of Physics, Queens College, City University of New York, Flushing, NY 11367 USA; 2 The Graduate Center of the City University of New York, New York, NY 10016 USA

**Keywords:** Metamaterials, Optical properties and devices, Nanophotonics and plasmonics

## Abstract

Many applications in environmental and biological sensing, standoff detection, and astronomy rely on devices that operate in the mid-infrared range, where active devices can play a critical role in advancing discovery and innovation. Nanostructured graphene has been proposed for active miniaturized mid-infrared devices via excitation of tunable surface plasmons, but typically present low efficiencies due to weak coupling with free-space radiation and plasmon damping. Here we present a strategy to enhance the light-graphene coupling efficiency, in which graphene plasmons couple with gold localized plasmons, creating novel hybridized plasmonic modes. We demonstrate a metasurface in which hybrid plasmons are excited with transmission modulation rates of 17% under moderate doping (0.35 eV) and in ambient conditions. We also evaluate the metasurface as a mid-infrared modulator, measuring switching speeds of up to 16 kHz. Finally, we propose a scheme in which we can excite strongly coupled gold-graphene gap plasmons in the thermal radiation range, with applications to nonlinear optics, slow light, and sensing.

## Introduction

Devices operating in the mid-infrared region of the electromagnetic spectrum find significant applications in imaging, sensing, communication, and astronomy due to the presence of unique molecular “spectral fingerprints”^1^ and atmospheric transparency windows between 3–5 μm and 8–12 μm^2^. Actively tunable optics targeting the mid-infrared would empower the development of these applications. Graphene is a material with optical and electronic properties that can be altered through the electrical injection of charges. In its charge-neutral form (undoped), free-standing graphene can absorb 2.3% of light across a wide range of frequencies due to interband transitions, as determined by the fine-structure constant^3^, and this universal absorption approximately holds for the mid-infrared range if undoped^4^. This absorption can be removed through electrically induced charge doping, as interband transitions are eliminated due to Pauli blocking as the Fermi energy increases^5^. Although this provides an impressive modulation capacity for a single-atom-thick material, for applications such as light modulators this value of 2.3% is insufficient^6^. Nevertheless, nanostructured graphene can couple to light through localized surface plasmons to enhance light absorption and scattering^7–11^, enabling greater optical absorption and modulation. Moreover, graphene plasmon resonances can be spectrally tuned across a significant part of the mid-infrared. The coupling, however, is inefficient due to wavevector mismatch between graphene plasmons and photons, the thinness of graphene, plasmon damping, and interaction with the substrate^8,9^. In addition, direct patterning of the graphene introduces defects at the edges which render the edges electrically inactive^9^. As a result, measured mid-infrared extinction rates in graphene nanostructures have been consistently smaller than 10%^7–9,12–15^, with a notable exception showing an extinction of 40% at high (0.8 eV) doping in a top-gated scheme with a high-capacitance ion-gel as the gate dielectric^14^.

Combining metallic structures with graphene has been explored for a variety of effects across the infrared and THz regimes^16–19^. More recently, alternative schemes have been proposed and demonstrated to dramatically enhance absorption in graphene by tailoring the interference between graphene-based plasmonic resonators and an optically separated reflecting backplane, forming a Salisbury screen^20–23^. Using such schemes, impressive modulation values can be achieved through electrical control of the graphene, as modulating the graphene properties sharply affects the interference condition between the plasmonic resonators and the backplane. A scheme using graphene slot resonators boosted the absorption modulation to ~ 25%^23^. Several other works used gold nanostructures on graphene, and such schemes have produced absorption values approaching 100% and modulation values of around 95%^20–22^. These other exciting results have showed the viability of using graphene as a platform for active mid-infrared optical elements and devices. However, these devices are limited to working in reflection only.

Here we propose an alternative approach, based on hybridization of gold-graphene plasmons. In this scheme, a strong near-field interaction between gold and graphene plasmons gives rise to tunable and sharp plasmonic resonances across the mid-infrared range. Gold plasmons serve as an intermediary between mid-infrared photons and deeply subwavelength graphene plasmons, enhancing the coupling between them. Additionally, the gold rods segment the graphene, effecting a nanoscale graphene pattern without etching of the graphene, thereby avoiding the creation of inert regions. With this approach, we demonstrate a metasurface in which a hybrid, graphene-like plasmon resonance can be tuned through the application of an external voltage, allowing for high differential transmission rates of 20% under moderate doping conditions and in atmospheric conditions. We also explore the potential application of such a device as an electro-optic modulator by applying an oscillating voltage source to the gate and measuring the optical modulation. Finally, we propose a design for a metasurface operating in the thermal radiation range showing strong coupling between graphene and gold plasmons, with potential applications in sensing and nonlinear optics.

## Results

### Hybridization model

**Figure 1:**
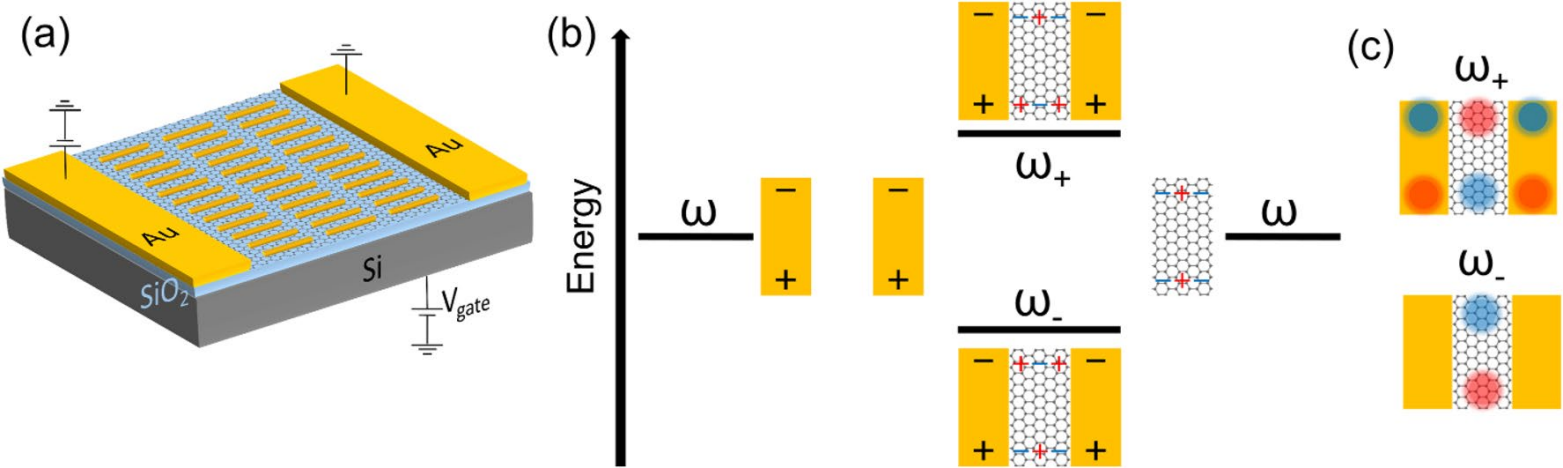
Hybridization between graphene and gold plasmons. (**a**) Design of the hybrid gold-graphene metasurface device. The thickness of the SiO_2_ dielectric spacer is 90 nm. The graphene layer is placed between the gold rods and the SiO_2_ spacer. (**b**) Microscopic depiction of the hybridization phenomenon. The incident light is polarized along the long (vertical) axis of the gold rod. The near-field around the gold rods couples to dark plasmons in graphene along the horizontal direction. The gold and graphene plasmons hybridize, forming two new modes, whose energies $\hbar {\omega }_{-}$ and $\hbar {\omega }_{+}$ depend on the charge distribution on graphene. (**c**) Net charge density of antisymmetric (${\omega }_{+}$) and symmetric (${\omega }_{-}$) modes. The red and blue clouds represent opposite charges.

The concept of hybridization in plasmonics was introduced by Prodan et al. to explain plasmon resonances in metallic nanostructures^24^. Originally applied to metallic nanoshells, it has been used to explain and design plasmon resonances in a myriad complex structures^25,26^, including in nanostructured graphene^13,27–29^. This powerful concept borrowed ideas from hybridization in chemistry to provide an intuitive picture to explain the plasmon resonances and visualize charge distribution. In our case, the metasurface design, depicted in Fig. 1a, will be based on hybridization between graphene and gold plasmons. It consists of gold nanorods deposited on a graphene surface in a periodic configuration. Mid-infrared light excites localized surface plasmons (LSPs) in the gold nanorods, which in turn launch graphene LSPs confined in the space between the tips of the gold rods. Previous experiments have demonstrated that graphene SPPs can be launched by scattering mid-infrared light from a particle near graphene^30,31^, and in this case a graphene LSP is generated due to the confinement of the graphene.

Due to the proximity of the graphene and gold LSPs, they couple through the near-fields, forming hybrid modes. We can control the detuning between graphene and gold LSP frequencies through their geometry and gate voltage. We will consider two cases separately: when these two resonances are far apart, and when they match. Figure 1b shows the concept for hybridization in our design. Graphene and gold plasmons will couple through the near-field, giving rise to two new hybrid modes, depending on their charge density configuration. In the *bonding* (symmetric) mode, the charge density will be concentrated mostly on graphene, while in the *antibonding* (antisymmetric) mode the charges will be more evenly distributed among the gold rods and graphene. The picture is equivalent to that of a coupled harmonic oscillator, but the hybridization concept provides a microscopic description of the coupling origin. The resonances will be calculated from electromagnetic simulations, including a model for each material’s permittivity or surface conductivity (see Supplementary Information).

In addition to near-field coupling, electric charges can move across the gold-graphene border since gold is in direct contact graphene. In the Supplementary Information, we discuss the effect of this charge movement to the spectrum and charge distribution for an analogous plasmonic system consisting of metallic nanorods. In our design, for the symmetric mode, the charges at the rod and on the graphene near the rod edge have opposite parity (${\omega }_{-}$, Fig. 1b). The electrical charges on graphene may spill under the rod, leading to a partial neutralization of the charge distribution under the rod. The amount of the remaining charges will depend, among other factors, on the charge density on graphene. If this charge density is sufficiently high, the charges moving across the border may overcome those under the rod, leading to a reversal of the sign of the charge. In contrast, for the antisymmetric mode (${\omega }_{+}$, Fig. 1b), the charge distribution at the rod and on graphene near the border have the same parity. Therefore, as the charges on graphene spill under the rod, the effect will be an increase in the charge density under the rod.

### Detuned resonances

**Figure 2:**
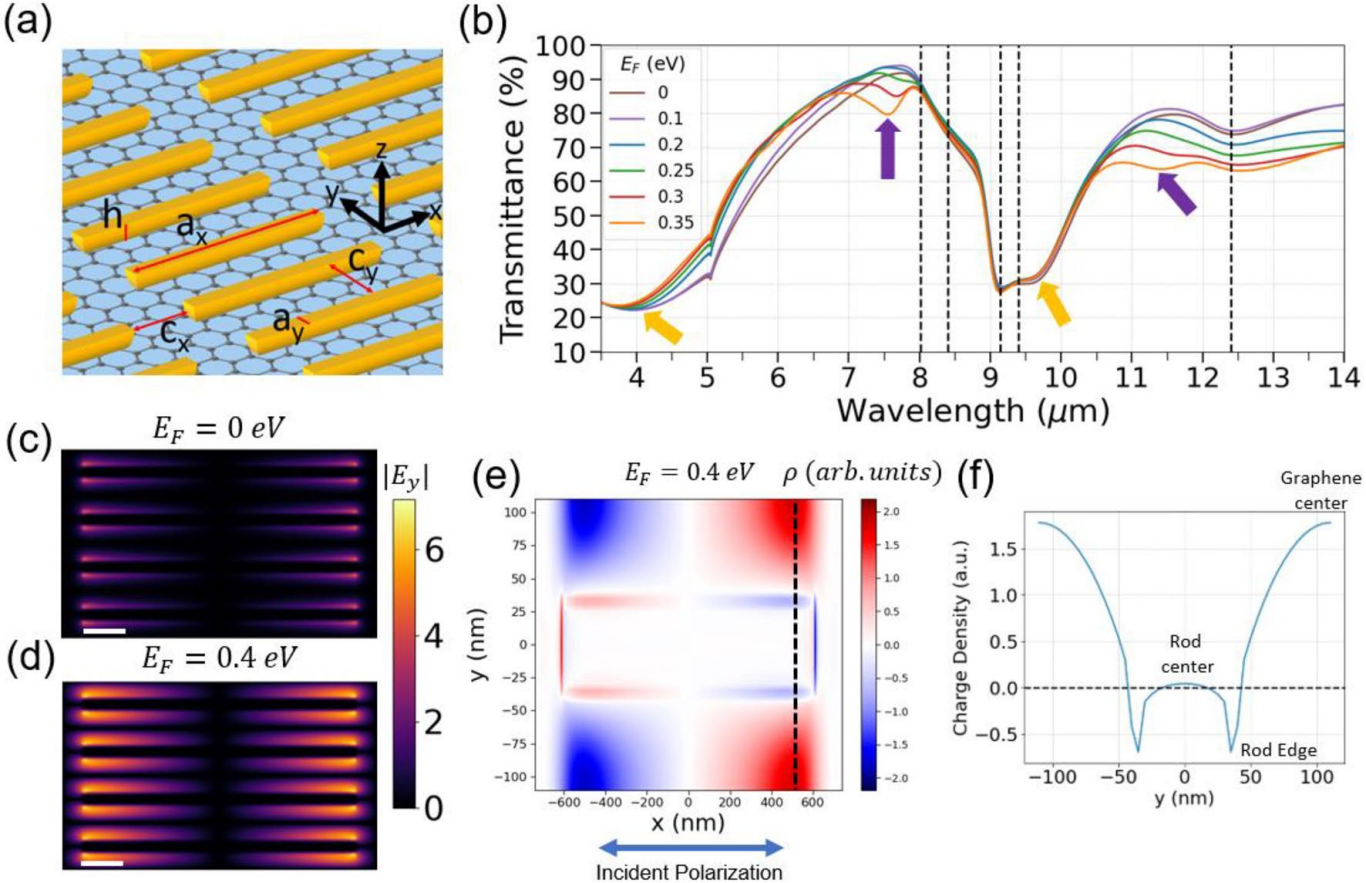
Design of the hybridized gold-graphene metasurface for detuned resonances. (**a**) Parameters of the metasurface: a_x_ = 1210 nm, a_y_ = 70 nm, c_x_ = 270 nm, c_y_ = 150 nm. The height *h* of the gold rods is 25 nm. Light is polarized along the x-axis (long axis of rods). (**b**) Calculated transmittance of the metasurface across the mid-infrared regime for various values of graphene’s Fermi level in eV. The gold and purple arrows depict “gold-like” and “graphene-like” resonances, respectively. Black dashed vertical lines indicate silica phonon lines. The damping parameter of graphene is 0.02 eV. (**c**) $|{E}_{y}|$ map at 0 eV (**d**) and at 0.4 eV. Color bar values represent field enhancement. Scale bar (white) is 200 nm. (**e**) Charge density map calculated at 0.4 eV. (**f**) Cross section of charge density at x = 520 nm, along the black dashed line in (**e**). All maps shown are calculated on the graphene plane with excitation wavelength λ = 7.5 µm, corresponding to a purple arrow in (**b**) and with horizontally (x-axis) polarized incident light. The charge density oscillations in (**e**) are mostly concentrated on the graphene area, and the resonance character is essentially that of graphene plasmons.

We begin by considering the scenario where the LSPs of both gold and graphene are detuned. The design is presented in Fig. 2a and the calculated spectra for varying graphene Fermi level are presented in Fig. 2b. The calculated spectra and fields are obtained using full-wave FDTD electromagnetic simulations, as described in the Methods section. When the Fermi level is set to 0 eV, the spectrum displays only a broad resonance of the gold rod, peaked at 4 µm and 9.6 μm. A small dip in the broad gold rod resonance appears at 5 μm, corresponding to a Wood’s anomaly mode at the interface of the silicon substrate, which is further elaborated on in the Supplementary Information. The additional features in the spectrum will be explained in the experimental section later. By adjusting the Fermi level of graphene, resonances centered around 7.5 µm and in the 11–13 µm range can be observed. The original gold LSP around 4 µm is also shifted by the coupling to the graphene LSP, though this effect is more limited. While the modes are hybridized, the graphene and gold LSPs generally retain their original character within the range of chemical potentials that we can access experimentally. Therefore, for detuned resonances, we will refer to these hybrid modes as *graphene-like* (purple arrows in Fig. 2b) and *gold-like* (gold arrows), respectively. Figure 2c,d provide a calculated map of the electric field magnitude in the vertical direction ($|{E}_{y}|$) at a graphene-like resonance ($\lambda$ = 7.5 µm) at Fermi energies of 0 eV and 0.4 eV, respectively, while Fig. 2e shows the charge density map at 0.4 eV. These maps demonstrate that this resonance is *graphene-like,* as the charge density oscillations (or electric fields) are concentrated on the graphene between the rod edges. Figure 2f shows a line profile of the charge density map taken at x = 520 nm, which shows a dark mode on graphene along the y-direction in accordance with Fig. 1b. The charge density is observed to be much larger on graphene than on the gold rods, which can be explained by two factors. First, the gold rod is excited far from resonance while the graphene dark mode is at peak resonance. Second, the transfer of opposite charge across the rod-graphene interface neutralizes some of the charge on the gold rod. Videos of the simulated charge density evolving over time (at 7.5 μm excitation) are available as Supplementary Video 1 (at 0 eV) and Supplementary Video 2 (at 0.35 eV).

**Figure 3:**
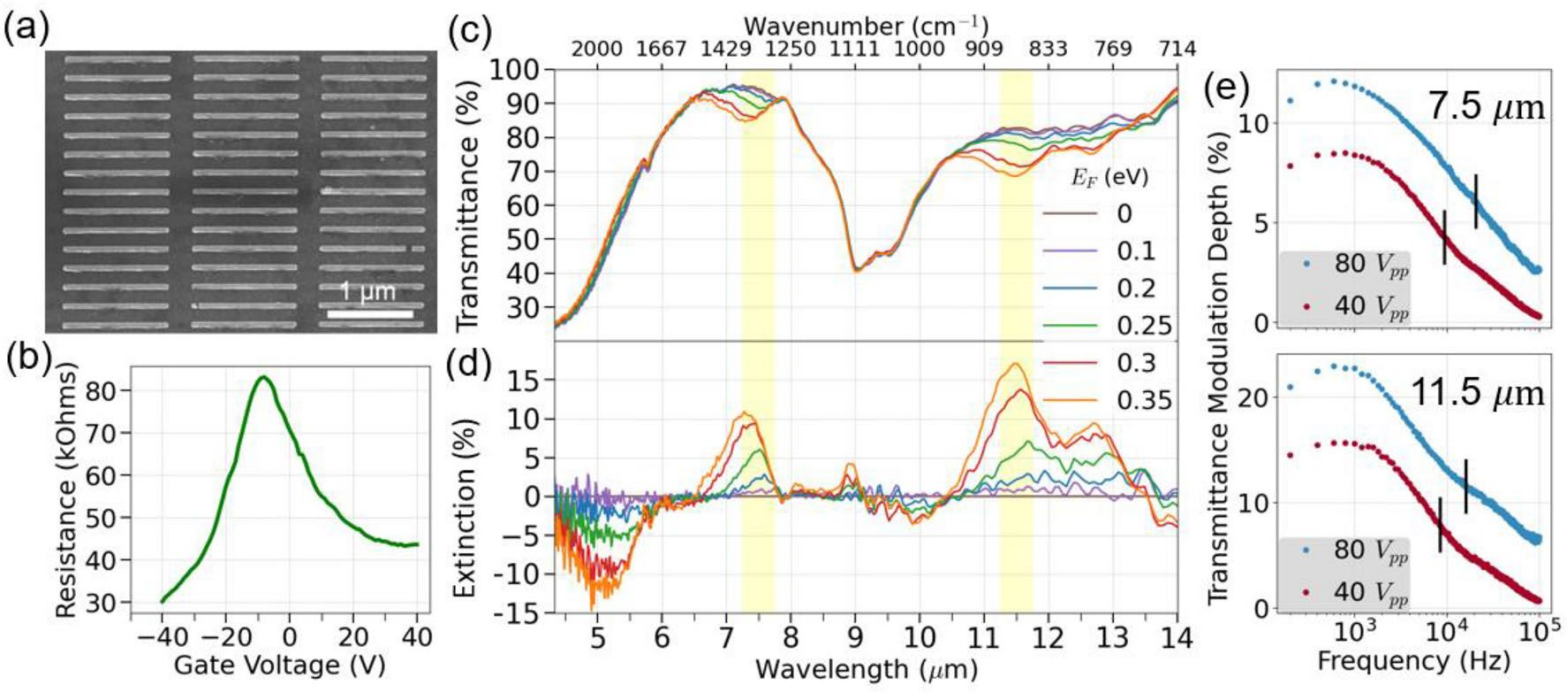
Characterization of the fabricated metasurface. (**a**) Scanning electron microscopy of a section of the device. (**b**) Transfer curve of the field-effect transistor device. (**c**) Measured transmittance as a function of graphene’s Fermi level, given in eV. (**d**) Calculated differential extinction. (**e**) Frequency response characterization of the mid-infrared modulator for sinusoidal gate voltages of 80 V (blue) and 40 V (red) peak-to-peak. The modulation experiment was conducted with an optical bandpass filter at 7.5 μm (top) and 11.5 μm (bottom), with passbands shown as the yellow bands in (**c**) and (**d**). Cutoff frequencies (− 3 dB, 50%) in (**e**) compared to max modulation) are marked with vertical black lines.

Figure 3a shows an SEM image of the fabricated metasurface, using e-beam lithography on a CVD-grown graphene field-effect transistor device^32^ (see methods section). The total area of the metasurface is 250 × 250 µm^2^. Figure 3b shows the measured transfer curve of the graphene device, which is used to determine the correspondence between the gate voltage and the graphene chemical potential. The spectral response of the fabricated device was measured using a Fourier Transform Infrared Spectrometer (FTIR) and is shown in Fig. 3c. The gate voltage was adjusted to tune the graphene chemical potentials from 0 to 0.35 eV. Figure 3d shows the differential extinction, defined here as $\Delta T\left({E}_{F}\right)=1-T\left({E}_{F}\right)/{T}_{CNP}$, where $T\left({E}_{F}\right)$ and ${T}_{CNP}$ are transmittances at the graphene’s Fermi level ${E}_{F}$ and at charge neutrality point (CNP, ${E}_{F}\hspace{0.17em}$= 0 eV), respectively. This metric compares the performance of our graphene metasurface at a given doping level to that of graphene at the CNP, and expresses the extent to which the transmission can be actively tuned. The FTIR data shows a strong differential extinction of 17% at 11.5 μm, which corresponds to an atmospheric window, and 11% at 7.5 μm. These two resonances correspond to a *graphene-like* plasmon. Some negative differential transmission can be seen where the *gold-like* LSP shifts, which results in higher transmission. The gold rod resonances peak around 4 and 9.6 μm, and coupling with the silica phonons^8,9^ in the 9.6 μm region results in a surface enhanced infrared absorption (SEIRA) effect^33,34^. A second peak around 12.8 μm is sensitive to the tuning of the graphene chemical potential, which is the result of splitting of the resonance due to the silica surface optical phonon around 12.4 μm^35^. The linewidths of the graphene-like LSPs are narrower than the gold LSPs due to the lower losses in graphene.

Additionally, we conducted an electro-optical modulation experiment by applying an alternating voltage source to the gate over a range of modulation frequencies while measuring the transmission modulation at a select wavelength. The wavelength was selected using a mid-infrared band-pass filter centered around 7.5 or 11.5 μm (500 nm FWHM bandwidth for both), and the measurements were taken with a lock-in amplifier. The transmission modulation data (Fig. 3e) shows strong modulation depths of up to 23% of 11.5 μm light and remains within 3 dB (50%) of the maximum modulation depth up to around 16 kHz. This demonstrates the device’s potential as an electro-optic modulator or as a laser noise eater. The sensing application is especially promising given that the device is operable in an atmospheric window while in contact with the ambient atmosphere and at room temperature. These results were achieved using CVD graphene on standard substrates (silica on silicon) that was processed through several rounds of lithographic processes, and thus represent a reasonable picture of such a device produced at scale.

The extinction spectrum of the device is significantly affected by the type of substrate used as well as the mobility of graphene. Our experiments showed that using a silica substrate, the device produced multiple resonances due to the dispersion of the refractive index, but the tunability of the spectrum was limited. To address this limitation, we conducted numerical simulations for Al_2_O_3_, which are presented in Supplementary Fig. 1. These simulations showed that by using Al_2_O_3_ substrate, we could tune the graphene resonances from 9 to 6.5 µm, providing greater spectral tunability. Additionally, we found that even with low mobility graphene, we were still able to achieve a significant response.

### In-tune resonances

We now focus our attention to the case where gold and graphene plasmonic resonances have the same frequency. Gold and graphene are materials with optimal plasmonic resonances that fall in different parts of the electromagnetic spectrum and are considered to be complementary^36^. While gold presents outstanding performance in the visible and near-infrared ranges, in the mid-infrared its optical extinction coefficient is too high and the resonances are broad. Graphene plasmons, on the other hand, can be fine-tuned through the THz and the mid-infrared down to around 6.4 µm, below which they are damped by graphene optical phonons^9^. Here, we will make use of gap plasmons to tune the gold rod resonance to the 8–10 µm window. The concept of gap plasmons has been thoroughly studied and documented^25,37–39^, and the mechanism for shifting the resonance to the mid-infrared is similar to that of hybridization^25^.

**Figure 4:**
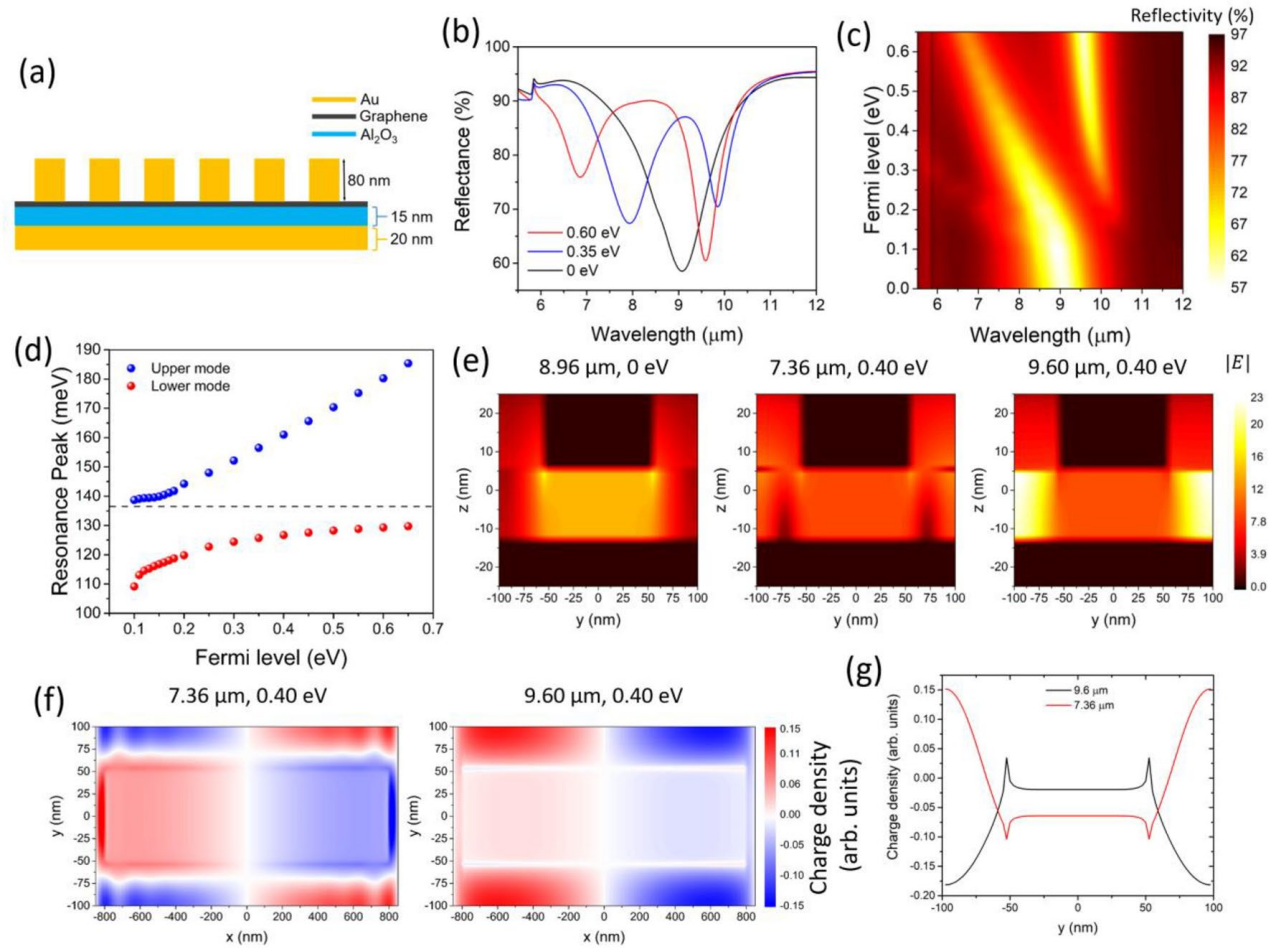
Design of hybridized gold-graphene metasurfaces for in-tune resonances. (**a**) Illustration of the metasurface’s cross-section. The parameters of the periodic array (top view shown in Fig. 2a) are a_x_ = 1600 nm, a_y_ = 110 nm, c_x_ = 90 nm, c_y_ = 100 nm. (**b**) Calculated reflectivity of the metasurface for three different values of graphene’s Fermi level, showing tunable symmetric and antisymmetric modes. The damping parameter of graphene is set to 13 meV (**c**) Reflectivity of the metasurface calculated for a larger range of graphene’s Fermi level. (**d**) Resonance peak for several Fermi levels. The minimum separation between the hybrid modes is at 0.18 eV, and corresponds to Δ = 23 meV. (**e**) Calculated near-field distribution of the gap-plasmons for E_F_ = 0 eV and λ = 8.96 µm (left), E_F_ = 0.40 eV and λ = 7.36 µm (center), and E_F_ = 0.40 eV and λ = 9.60 µm (right). The field was calculated at the position x = 700 nm. (**f**) Calculated charge density map (arbitrary units) on the graphene plane for E_F_ = 0.40 eV and λ = 7.36 µm (left), E_F_ = 0.40 eV and λ = 9.60 µm (right). (**g**) Cross-section of the charge density along the y-axis at x = 650 nm for both the upper and lower modes.

The design is shown in Fig. 4a. The gold rods are separated from a gold plane by a thin 15 nm dielectric (Al_2_O_3_) layer and graphene. When coupled to light, the rods induces mirror charges on the metal plane beneath it, and the plane and the rods couple. As a result, the bonding mode arising from this interaction falls in the mid-infrared around 9.1 μm, as seen in the spectrum in Fig. 4b for a graphene Fermi level of 0 eV. By increasing the Fermi level of graphene (Fig. 4b,c), graphene plasmons are excited and hybridization between graphene-gap and gold-gap plasmons takes place. We can observe the splitting of the two hybridized modes, which is clearly seen by plotting the peaks as a function of Fermi level in Fig. 4d, and the near-field images and charge density distribution shown in Fig. 4e,f respectively are in line with the hybridization picture in Fig. 1. By using doping as the detuning parameter, we observe an anti-crossing behavior, and at the point of minimal detuning (*E*_F_ = 0.18 eV), the separation between the hybrid modes is $2g = 23.0$ meV. This simple examination of the coupling may underestimate the true coupling strength, see Supplementary Information for details.

We compare this coupling value to the widths of the resonances of the hybrid modes ${y}_{+}\hspace{0.17em}$= 24.9 meV and ${y}_{-}$ = 8.3 meV. In this design, the coupling rate $2g>({y}_{+}+{y}_{-})/2$, indicating the system is in the strong coupling regime. In this regime, the two modes exchange energy at a rate faster than their dissipation rate, resulting in longer-lived plasmons and narrowed resonances. Accordingly, the linewidths ${y}_{+}$ and ${y}_{-}$ of both hybrid modes at ${E}_{F}=0.4 {\text{eV}}$ are smaller than that of the unhybridized mode, ${y}_{0}$ = 26.6 meV, calculated at ${E}_{F}=0 {\text{eV}}$.

Finally, as we increase the Fermi level, the charge density distribution is still concentrated on graphene for the bonding mode (*graphene-like*, right panel of Fig. 4f), as expected by the hybridization picture provided in Fig. 1c. For the antibonding mode however, the charge density is distributed on gold and graphene (left panel of Fig. 4f), assuming a truly hybrid character. In the left charge density map of Fig. 4f, we can observe a spatial modulation of the charge density. This is caused by propagating graphene plasmons, which are launched by the gold rods along the x-axis, and interfere with the localized plasmons between the rods (along the y-axis). Figure 4g shows a line profile of the charge density maps of Fig. 4f taken at x = 650 nm for both the bonding and antibonding modes. The line profile is in agreement with the microscopic picture illustrated in Fig. 1b,c. For the bonding mode, the charge density on the gold rod is much weaker than that on the graphene center due to transfer of charge across the gold-graphene interface which partially neutralizes the charge under the rod. For the antibonding mode, the charges on the graphene center have opposite polarity to the charges on the center of the gold rod and at the edge. In both cases, a dark mode can be observed on the graphene along the y-axis between the gold rod edges.

## Discussion and summary

We have presented a simple design that creates a hybridization between graphene and gold plasmons, offering a microscopic view of their interaction and enabling the creation of more sophisticated structures in the future to improve field localization. Using a commercially available, large-area CVD-grown graphene field effect transistor, we have demonstrated narrow resonances in the mid-infrared region, achieving up to 17% transmission modulation with only a modest 0.35 eV chemical doping. Our device also showed dual band modulation of 20% at a (3 dB) rate of up to 16 kHz, with potential improvements in switching speed by reducing the device area and consequently the device’s sheet resistivity.

The tunable, narrow resonances of our design make it highly desirable for mid-infrared metasurfaces. A metasurface is composed of an array of subwavelength particles on a surface that can couple with incident light^25,40–42^. The geometry of each particle in the array can be designed to alter the amplitude, phase, and polarization of the incident light at a subwavelength scale, which can effect such functionalities as lensing^43,44^, beam-steering^45^, holography^46^, and nonlinearity^47–50^. Our metal-on-mirror design with matched resonances offers two distinct regions where the amplitude and phase of mid-infrared radiation can be controlled. Moreover, the sharp dip at the center of the spectrum resembles an electromagnetic-induced transparency dip that could be used to observe electrically tunable “slow light”^51^ in the mid-infrared range.

Since our design employs localized plasmonic modes instead of propagating modes, the microscopic picture in Fig. 1 may be applied to single hybrid structures (as illustrated in Fig. 2b,c), opening up the possibility of performing true single particle measurements with tunable graphene-gold plasmons. The hybridization can also be extended to non-periodic arrangements, offering larger flexibility in terms of metasurface design.

The device's extinction rate can be improved by optimizing parameters such as the dielectric gate material, dielectric thicknesses, array dimensions, and index matching between the top and bottom surfaces. Calculations for a metasurface using Al_2_O_3_ as the gate dielectric are presented in the Supplementary Information and showed enhanced extinction rates. When searching for the best parameters, either rational (from hybridization theory) or inverse design methods^52^ can be applied, or even a combination of both.

The fact that our design is exposed to air may greatly benefit applications such as biosensing^53^ and gas detection^54^. Moreover, the multiple resonances can enhance coherent anti-Stokes Raman signals. Finally, one can visualize applications such as tunable mid-infrared band-pass filters, active control of thermal radiation and cooling.

## Materials and methods

### Numerical simulations

The design of the device was carried out using the Lumerical FDTD package, using a graphene conductivity model^55^. The optical constants of silicon, gold, chromium, SiO_2_ and Al_2_O_3_ were taken from Palik handbooks^56^.

The simulations were carried out with a broadband pulsed plane wave illumination source (2 to 16 µm) under normal angle incidence with polarization along the long axis of the gold rods. Perfectly matched layers were used at the z-limits of the simulation to reduce reflections, and symmetric periodic boundary conditions were used along the xy-plane, as the designed structure is symmetric about both axes.

See the Supplementary Information for more details.

### Device fabrication

A GFET S11 from Graphenea was used. It is a commercially produced chip of CVD graphene transferred to a 90 nm silica layer on top of p-doped silicon. Graphenea creates the microstructure of graphene patches in contact with gold pads using photolithography. The mobility of graphene was 2472 cm^2^/V∙s. We then created the metasurfaces on the graphene patches through an e-beam lithography process. Bilayer PMMA was spun on and exposed with an e-beam to form a deposition mask. The bottom layer is 2% solids 495K molecular weight PMMA in anisole solution (Kayaku PMMA 950K A2), and the top is 4% solids 950K molecular weight PMMA in anisole solution (Kayaku PMMA 950 A4). Both layers were individually spun at 4000 RPM with a 500 RPM/s ramp for 60 s, each spin followed by a bake at 180 C for 90 s. The resist was exposed with an Elionix 50 keV tool with a beam current of 200 pA using a shot pitch of 2 nm and 0.184 μs per shot. The deposition mask was developed in a 1:3 dilution of MIBK in IPA (60 s) followed by an IPA bath (30 s). 3 nm of Cr (adhesion layer, 0.3 Å/s) followed by 25 nm of Au (0.5 Å/s) was deposited through the mask to form the gold rods of the metasurface. A liftoff process with acetone was employed to obtain the clean metasurface seen in Fig. 3a. The GFET S11 was attached to a printed circuit board, and the gold pads were wire-bonded using an electrically conductive epoxy (EPO-TEK, H20E).

### Optical and electrical characterization

The transmittance spectra of the device is obtained in a home-built infrared microscope using a Fourier Transform Infrared Spectrometer (Newport MIR8035) coupled to a Mercury Cadmium Telluride detector (Newport 80,026 MCT). The resolution of the FTIR spectrometer was set to 8 cm^−1^. A broadband SiC mid-infrared light source (Newport 80,007 SiC) is focused on the sample using a gold parabolic mirror to a spot size comparable to the metasurface array. A ZnSe focusing lens collects the transmitted light and directs it on to either an infrared camera (for targeting) or to the FTIR (for spectral analysis) using a motorized flip mirror. A spectrum is also taken from a nearby section of silica/silicon without a graphene metasurface for the purposes of normalization of the spectrum to the SiC light source. A ZnSe wire grid linear polarizer (Thorlabs, WP25H-Z) is placed after the microscope to transmit infrared light polarized along the long axis of the gold rods. The thermal background with the signal blocked is also taken so that the thermal background can be removed from the data.

The chemical potential of the graphene is adjusted by applying a voltage to the silicon backgate of the device (Keithley 2400) while measuring the source-drain current at a source-drain voltage of 1 mV (Keithley 2401). Using these source-measure units, the transfer curve of the graphene device was taken. The graphene intrinsic doping slowly but noticeably shifts as a gate voltage is applied to it, which manifests as a changing source-drain current. During the experiment, a target current, which corresponds to a desired chemical potential, is selected, and the gate voltage is adjusted in real time to maintain that target current.

### Mid-infrared radiation modulation

The frequency modulation data is obtained via the same optical setup as the transmittance measurement, but instead the light was routed directly to the MCT detector, without passing through the FTIR. Mid-infrared bandpass filters centered at 7.5 µm (Edmund Optics, #17–247) and 11.5 µm (Thorlabs, FB11500-500) were used to select different resonances of the metasurface. The gate voltage was modulated using an arbitrary waveform generator (Keithley 3390) and a voltage amplifier (Thorlabs, HVA200) to provide a sinusoidal high-voltage to the gate. The root mean squared voltage from the MCT detector was measured using a 200 kHz lock-in amplifier (Stanford Research Systems, SR 830). The MCT detector responsivity rolls off below 200 Hz.

### Supplementary Information

Supplementary Information.

Supplementary Video 1.

Supplementary Video 2.

## Data Availability

Data supporting this work are available upon request from the corresponding author.
