# Supplementary material for: Hybridization of graphene-gold plasmons for active control of mid-infrared radiation
Source: Sci Rep. 2024 Mar 20;14:6733. doi: 10.1038/s41598-024-57216-6 (PMC10954650; doi:10.1038/s41598-024-57216-6)
Supplement: Supplementary file 1 — Supplementary Information. [file 41598_2024_57216_MOESM1_ESM.pdf]

Supplementary information for

Hybridization of Graphene-Gold Plasmons for Active Control of Mid-Infrared Radiation

Matthew D. Feinstein[1,2], Euclides Almeida*[1,2]

[1]*Department of Physics, Queens College, City University of New York, Flushing, NY 11367, United States of America*

[2]*The Graduate Center of the City University of New York, New York, NY 10016, United States of America*

**Author's email address: euclides.almeida@qc.cuny.edu*

Supplementary Video 1

Simulated time evolution of the charge density on the metasurface under 7.5 μm excitation (~120 fs pulse duration) and at graphene Fermi energy of 0 eV. The tail of the gold rod resonance (centered at 5 μm) is weakly excited, but there is little charge oscillation on the graphene since there is no net charge.

Supplementary Video 2

Simulated time evolution of the charge density on the metasurface under 7.5 μm excitation (~120 fs pulse duration) and at graphene Fermi energy of 0.35 eV. The tail of the gold rod resonance (centered at 5 μm) is weakly excited, which in turn launches plasmons on the charged graphene and establishes a standing wave between the gold rod tips in the vertical direction. The transient response shows a standing wave in the horizontal direction as well.

Numerical Simulation Details

In our numerical simulations, used the 2D graphene conductivity model was used for graphene [1]. This model calculates the optical conductivity as composed of the intraband (σ_{intra}) and interband (σ_{inter}) conductivity terms:

$$\sigma(\omega) = \sigma_{intra}(\omega) + \sigma_{inter}(\omega)$$

The intra and interband conductivities are given by:

$$\sigma_{intra}(\omega) = \sigma_{intra}(\omega, \mu, \Gamma, T) = \frac{ie^2}{\pi\hbar^2(\omega - i2\Gamma)}\int_0^\infty \varepsilon\left(\frac{\partial f(\varepsilon)}{\partial\varepsilon} - \frac{\partial f(-\varepsilon)}{\partial\varepsilon}\right)d\varepsilon$$

$$\sigma_{inter}(\omega) = \sigma_{inter}(\omega, \mu, \Gamma, T) = \frac{ie^2(\omega - i2\Gamma)}{\pi\hbar^2}\int_0^\infty \frac{f(-\varepsilon) - f(\varepsilon)}{(\omega - i2\Gamma)^2 - 4(\varepsilon/\hbar)^2}d\varepsilon$$

Where μ is the chemical potential, Γ is a phenomenological (graphene) damping parameter, T is the temperature, e is the elementary charge, $f(\varepsilon) = \frac{1}{\exp[(\varepsilon-\mu)/k_B T]-1}$ is the Fermi-Dirac distribution. k_B is the Boltzmann constant. In the simulation of our fabricated metasurface, the phenomenological parameter was set to 0.02 eV. This model does not take into account plasmon damping by graphene phonons below 6.3 μm, and our calculations were restricted to graphene plasmons at longer wavelengths.

Estimation of the Coupling Strength

In our assumptions in the main text, we slightly underestimate the coupling strength between gold and graphene plasmons when we use the minimal detuning point to find $2g = 23.0\ meV$. This is because tuning graphene's Fermi level also changes the damping rate of graphene plasmons. Specifically, at lower doping the interband damping is more pronounced, which inhibits the coupling strength. the coupled harmonic oscillator model, the frequency of the hybrid (normal) modes, are given by [2,3]

$$\omega_{\pm} = \omega_0 - i\left(\frac{\gamma_{Au}}{2} + \frac{\gamma_{gr}}{2}\right) \pm \frac{1}{2}\sqrt{\Omega^2 - \left(\frac{\gamma_{Au}}{2} - \frac{\gamma_{gr}}{2}\right)^2} \quad (1)$$

where in our case, ω_0 is the resonance frequency at E_F = 0 eV, γ_{Au} and γ_{gr} are the damping rates of gold and graphene plasmons respectively, and Ω is the gold-graphene plasmons interaction potential ($\Omega = 2g$ for $\gamma_{Au} = \gamma_{gr}$). Therefore, the frequency of the hybrid modes should be equidistant to ω_0. This occurs at E_F = 0.27 eV, in which ω_+=149.7 meV, ω_- = 123.5 meV, and the coupling rate $2g$ = 26.2 meV.

Simulations on Different Substrate: Alumina

Simulations of a similar device on an Alumina substrate but without any reflecting backplane was carried out to demonstrate the spectral tunability of our device on a less dispersive substrate than silica. The results are shown in Supplementary Figure 1.

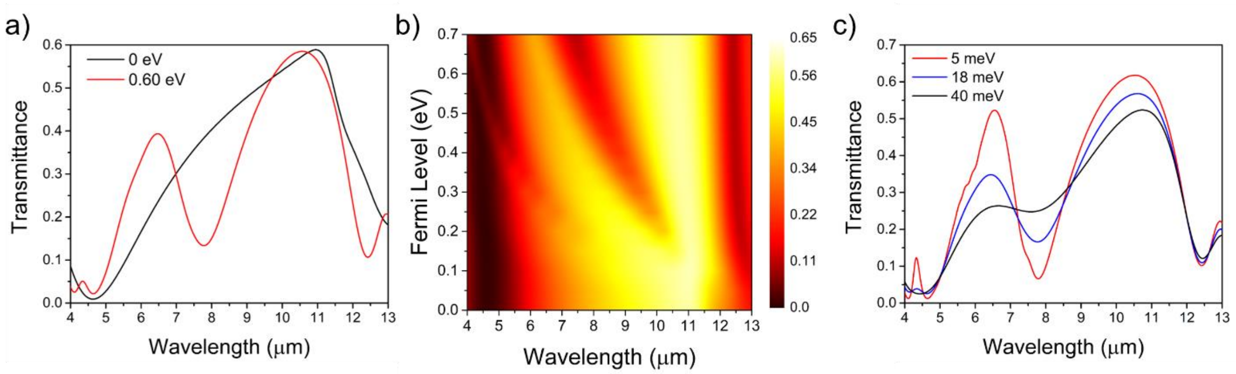

Supplementary Figure 1. Simulation of the hybrid metasurface using Al_2O_3 gate dielectric on silicon. A) Calculated transmittance of the metasurface for graphene's Fermi level E_F = 0 eV (black curve) and E_F = 0.60 eV (red curve). The parameters of the array are (top view shown in Fig. 2(a): ax = 900 nm, ay = 60 nm, cx = 150 nm, cy = 100 nm. The height of the gold rods is h = 20 nm, and the thickness of the Al_2O_3 spacer is t = 15 nm. The damping parameter of graphene is 13 meV. b) Calculated transmittance of the metasurface for various E_F. c) Calculated for graphene's damping parameter 5 meV (red), 18 meV (blue) and 40 meV (black). In this simulation, E_F = 0.6 eV.

Plasmonic hybridization for electrically coupled nanostructures

Most hybridization models present in the literature discuss plasmonic structures with small gaps. Therefore, the structures couple only through near-field interaction. In our system, we present an extreme case, where the gap between structures (gold rods and the connecting graphene) is zero. As we will see below, our structures still hybridize, since the electrical charges on the gold nanorods and on graphene oscillate in orthogonal directions. To illustrate plasmonic coupling (and hybridization) in the gapless case, we show in Suppl. Fig. 2(a) FDTD calculations for a familiar system where "plasmonic induced transparency" (PIT) occur (Phys. Rev. Lett. 101, 047401, 2008). In this "dolmen" structure, the incident electric field is polarized along the long axis of the top horizontal rod, which couples with the pair of vertical rods at the bottom for small gaps. This structure hybridizes, where the original plasmonic resonance is split into two modes of different charge density configurations. As shown in Suppl. Fig. 2(b), the energy splitting between the modes increases as the gap decreases. For the extreme gapless case, the two hybrid modes are still present and the splitting reaches its maximum. The extreme case in PIT also occurs if the structures are comprised of different materials, just like in our case, as shown in Suppl. Figs 2(d-f) for silver-gold rods coupling.

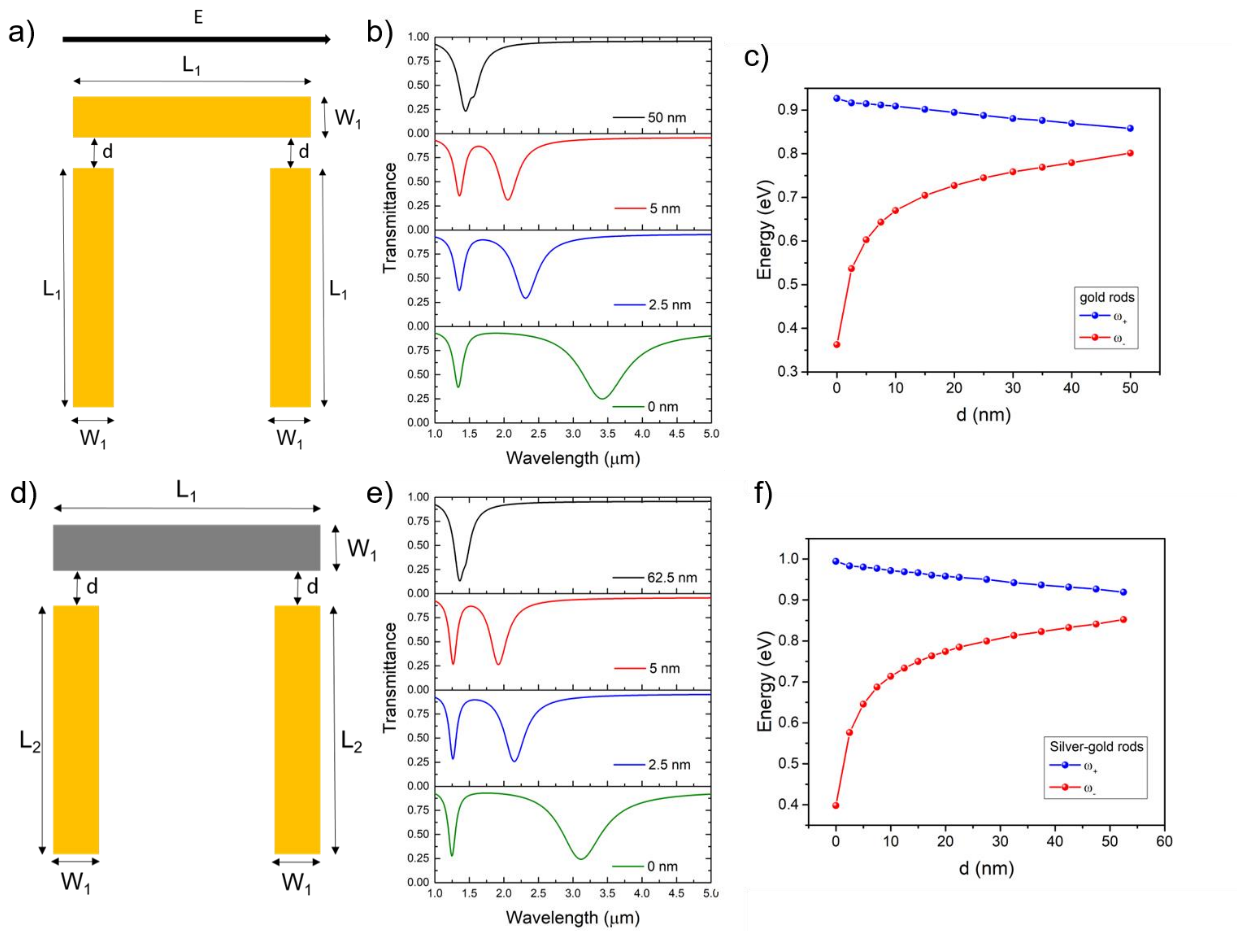

Supplementary Figure 2. Plasmonic coupling and hybridization for electrically connected nanorods. a) Geometry under consideration, where we show a unit cell of a periodic array of gold rods on a substrate with refractive index n=1.5. The incident light (E) is polarized along the long axis of the top rod. The parameters are L1 = 300 nm, W1 = 60 nm. The array periodicity is px=640 nm and py=500 nm. The thickness (height) of the rods is 20 nm. b) Transmittance spectrum for a) calculated for d = 50, 5, 2.5 and 0 nm (black, red, blue and green curves, respectively). At d = 0 nm, the two coupled modes are still present. c) Calculated peaks for the antisymmetric (blue balls) and symmetric (red balls) modes. d) Same geometry as a), but with a silver rod at the top. Here, L2 = 275 nm. e) Transmittance spectrum for b) calculated for d = 62.5, 5, 2.5 and 0 nm (black, red, blue and green curves, respectively). As in the geometric arrangement in a), at d = 0 nm, the two coupled modes are still present.

Hybridization between dark and bright modes in electrically connected plasmonic nanorods

To exemplify how we arrived at the dark-mode charge configuration in Fig. 1(b) and the illustration of the charge distribution in Fig. 1(c), we present in Supplem. Fig. 3(a) a modification of the structure show in Supplem. Fig. 2(d). We add another silver rod at the bottom and change the rods dimensions. This structure is similar to our graphene-gold geometry, where the silver rods play the role of our gold rods with a dipolar excitation, while the vertical gold rods play the role of graphene. For a proper choice of dimensions, a dark mode can excited on the connecting gold rods (Supplem. Fig. 3(c,d)). The transmittance spectrum is shown in Supplem. Fig. 1(b) and the charge density distributions are show for d = 12.5 nm (Supplem. Fig. 3(e,f)) and d = 0 nm (electrical contact, Supplem. Fig. 3(g,h)). We can clearly observe the dark mode excitation on the gold rods (+-+ and -+-) for the symmetric (ω_-) and antisymmetric (ω_+) modes at d = 12.5 nm. When the rods are electrically connected (d=0), for the symmetric mode, there will be a partial neutralization of the electrical charges at the junctions, since the charges have opposite parity at the rods end (Supplem. Fig. 1(h)). For the antisymmetric mode, however, the charges have the same parity, leading to an increase of the charge density at the junctions. (Supplem. Fig. 1(g)).

Wood's Anomaly at the Silicon-Metasurface Interface

In the simulated transmittance spectra of Fig. 2b, a slight dip inside the broad gold resonance can be seen around 5 μm. We attribute that effect to a Wood's anomaly, which for zero degree incidence can be expected to have a wavelength calculated by

$$\lambda = \frac{p}{m} \times n$$

where *p* is the periodicity of the structure, *m* is the mode, and *n* is the refractive index of the substrate. The relevant periodicity is that along the long axis, p = 1.48 μm, as the short axis periodicity of 340 nm produces an effect outside of the mid-infrared. . For the metasurface-air interface, the first Wood anomaly occurs at a wavelength matching the (long-axis) structure periodicity, λ = 1.48 μm, which is outside the spectral region of interest under consideration. At the silicon-metasurface interface, however, the first Wood anomaly occurs at 5 μm, taking the refractive index of silicon n = 3.43 used in our simulations, and this appears as a small dip in the transmittance.

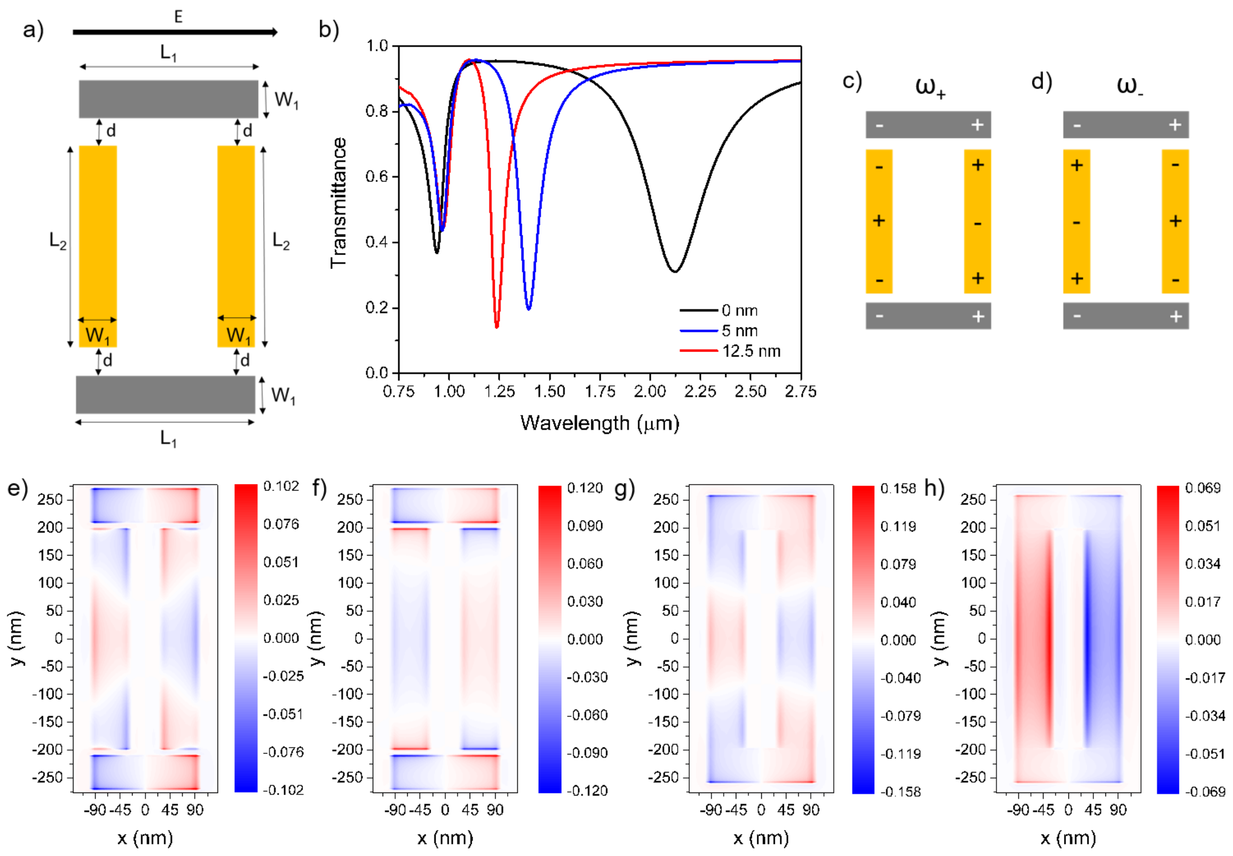

Supplementary Figure 3. Hybridization between dark and bright modes in electrically connected plasmonic nanorods. a) Geometry under consideration. The parameters are L1 = 200 nm (silver rod length), L2 = 400 nm (gold rod length), and W1 = 60 nm. The height of the rods is 20 nm. The array periodicity is Px = 400 nm and Py = 800 nm. b) Transmittance spectrum for d = 12.5 nm (red curve) and d = 0 nm (black curve). c,d) Charge configurations for the antisymmetric (c) and symmetric (d) modes. (e-h) Charge density distribution (arbitrary units) for $\lambda = 0.965$ μm, d= 12.5 nm (e); $\lambda = 1.25$ μm, d= 12.5 nm (f); $\lambda = 0.937$ μm, d= 0 nm (g), and $\lambda = 2.2$ μm, d= 0 nm (h).

In Supplem. Fig. 4(a), we show the transmittance spectra of the metasurface by replacing the silicon substrate with a dielectric of varying refractive index. In Supplem. Fig. 4(b), the Wood anomaly peak wavelengths are plotted against the refractive index of the dielectric substrate. The Wood anomaly dip shifts with the refractive index, according to the equation above. It is worthwhile to note that the Wood anomaly spectrum appears asymmetric. This Fano-like shape is likely caused by interference between the broad gold

rod plasmon resonance with the sharp Wood anomaly. The strength of the Wood anomaly dip increases as the spectral overlap with the gold rod resonance improves.

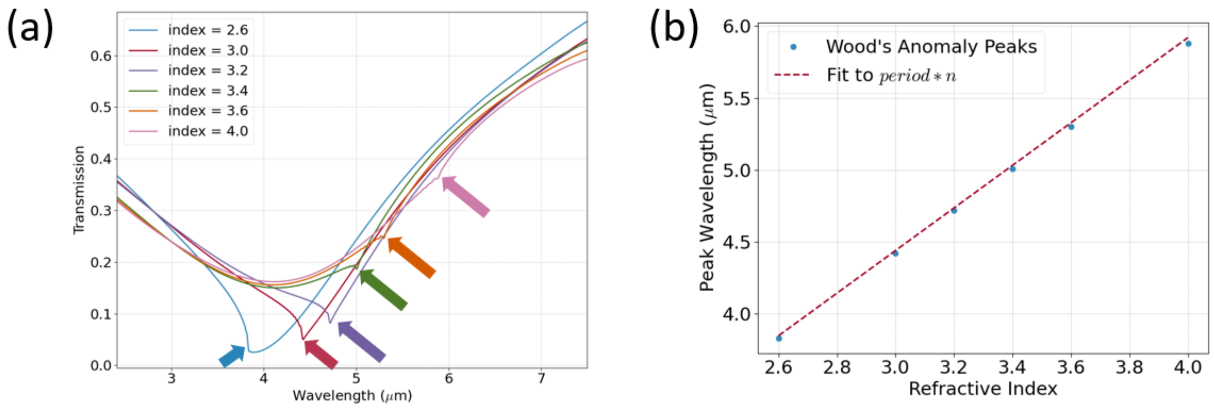

Supplementary Figure 4. Wood's Anomaly at Metasurface-Silicon interface. a) Transmission spectra of the gold rod resonance with the silicon substrate replaced by a dielectric of varying refractive index. Arrows point to the Wood anomaly feature for each spectrum. b) Peak wavelengths of the anomaly from (a) plotted against the refractive index of the substrate (blue circles), along with the fit to $\lambda = p \times n$ (red dashed line). For the above calculations, the graphene Fermi energy was set to 0 eV.

References

[1] G. Hanson, Journal of applied physics **103**, 064302 (2008).

[2] P. Törmä and W. L. Barnes, RoPP **78**, 013901 (2015).

[3] V. Savona, L. C. Andreani, P. Schwendimann, and A. Quattropani, Solid state communications **93**, 733 (1995).
